# How Much Lowering of Blood Pressure Is Required to Prevent Cardiovascular Disease in Patients With and Without Previous Cardiovascular Disease?

**DOI:** 10.1007/s11886-022-01706-4

**Published:** 2022-05-07

**Authors:** Dexter Canoy, Milad Nazarzadeh, Emma Copland, Zeinab Bidel, Shihir Rao, Yikuan Li, Kazem Rahimi

**Affiliations:** 1 Deep Medicine, Nuffield Department of Women’s and Reproductive Health, University of Oxford, Hayes House 1F, 75 George St, Oxford, OX1 2BQ UK; 2 National Institutes of Health Research Oxford Biomedical Research Centre, Oxford University Hospitals NHS Foundation Trust, Oxford, UK

**Keywords:** Hypertension, Anti-hypertensives, Therapeutics, Randomised controlled trials, Meta-analysis, Cardiovascular diseases

## Abstract

**Purpose of Review:**

To review the recent large-scale randomised evidence on pharmacologic reduction in blood pressure for the primary and secondary prevention of cardiovascular disease.

**Recent Findings:**

Based on findings of the meta-analysis of individual participant-level data from 48 randomised clinical trials and involving 344,716 participants with mean age of 65 years, the relative reduction in the risk of developing major cardiovascular events was proportional to the magnitude of achieved reduction in blood pressure. For each 5-mmHg reduction in systolic blood pressure, the risk of developing cardiovascular events fell by 10% (hazard ratio [HR] (95% confidence interval [CI], 0.90 [0.88 to 0.92]). When participants were stratified by their history of cardiovascular disease, the HRs (95% CI) in those with and without previous cardiovascular disease were 0.89 (0.86 to 0.92) and 0.91 (0.89 to 0.94), respectively, with no significant heterogeneity in these effects (adjusted *P* for interaction = 1.0). When these patient groups were further stratified by their baseline systolic blood pressure in increments of 10 mmHg from < 120 to ≥ 170 mmHg, there was no significant heterogeneity in the relative risk reduction across these categories in people with or without previous cardiovascular disease (adjusted *P* for interaction were 1.00 and 0.28, respectively).

**Summary:**

Pharmacologic lowering of blood pressure was effective in preventing major cardiovascular disease events both in people with or without previous cardiovascular disease, which was not modified by their baseline blood pressure level. Treatment effects were shown to be proportional to the intensity of blood pressure reduction, but even modest blood pressure reduction, on average, can lead to meaningful gains in the prevention of incident or recurrent cardiovascular disease.

## Introduction

Raised blood pressure is the world’s leading cause of premature death [1, 2]. Over 1.13 billion people globally have elevated blood pressure with two-thirds of them living in low- and middle-income countries; yet fewer than 1 in 5 people have their blood pressure levels under control [3]. Indeed, decreasing the prevalence of elevated blood pressure globally is one of the key targets of the World Health Organization’s Global Action Plan for 2013 to 2020 to reduce premature cardiovascular disease (CVD) deaths by 2025 [4]. Modifying this risk through pharmacologic lowering of raised blood pressure also plays an important strategy to reduce the burden of CVD. Findings from several randomised controlled trials (RCT) conducted in the past few decades across different countries have shown that pharmacologic lowering of blood pressure is effective in reducing the risk of CVD [5•, 6–12]. These beneficial effects have been reported to be consistent across major antihypertensive drug classes, and the relative effects on cardiovascular outcomes are broadly proportional to the magnitude of blood pressure reduction [5•, 13]. Despite the existing and robust evidence on the efficacy of blood pressure–lowering treatment, major controversies remain particularly with regards to treatment strategies, such as the appropriate blood pressure threshold to start pharmacologic treatment, the blood pressure to target when on treatment, and whether these strategies should differ depending on an individual’s history of CVD and baseline blood pressure. These uncertainties have been reflected in differences in certain aspects of clinical guidelines in the management of raised blood pressure. [14–16].

In this paper, we explore some of the earlier findings that have established the importance of raised blood pressure as a risk factor of CVD and describe observations that gave rise to uncertainties in the management of raised blood pressure. We will show reports demonstrating the efficacy of blood pressure–lowering drug treatment, and highlight findings from recent studies that examined the efficacy across strata of baseline blood pressure in people with and without prior CVD. We will then discuss how recent evidence addressed uncertainties in blood pressure–lowering treatment strategies. Finally, we will discuss and show future directions that can help inform clinical management of people with raised blood pressure.

## Association Between Blood Pressure and Cardiovascular Disease Risk: Epidemiological Observations

Observations from prospectively followed cohorts of community-dwelling persons, who were usually free of known cardiovascular disease, have informed the importance of elevated blood pressure as a risk factor of vascular conditions. Investigations from the Framingham Study, which originally recruited 5209 men and women aged 30 to 62 years in 1948 who were then prospectively followed, have helped demonstrate the relationship between elevated blood pressure and the risks of incident coronary heart disease and stroke [17•, 18•, 19, 20]. While findings from the Framingham Study have contributed to establishing raised blood pressure as a risk factor of CVD, the size of the study was too small to allow reliable characterisation of the risk across a wide range of blood pressure level. Several decades after the Framingham Study began, other population-based cohorts were formed, which helped to refine our understanding on the impact of elevated blood pressure on vascular disease risk. By combining data from several of these prospective studies, robust findings supported the earlier observations indicating that, in people without prior CVD, raised blood pressure increases the risk of CVD [21•]. This association was seen across the population range of blood pressure, without any evidence that the risk is limited to those with high values, such as in hypertension, nor was there any observed elevation in the risk in the lower end of the population distribution of blood pressure. These findings have been extended by the Prospective Studies Collaboration by showing that this association is consistent across middle to older age groups [22•]. This pattern in the association is not limited to cohort studies with standardised clinical measurements. Using data extracted from routine clinical practice settings has shown similar findings [23•]. In these large-scale prospective studies, there has been no blood pressure level below which the association with CVD risk is no longer valid. That is, there is a continuous gradient in the risk of CVD with increasing blood pressure level; conversely, CVD risk falls with lower blood pressure levels.

In contrast, observations of the association between blood pressure and CVD seem to be different in people who have had a prior vascular disease [24, 25]. For example, in patients with a stable coronary artery disease, the risk of developing a composite outcome of cardiovascular death, myocardial infarction, or stroke has been reported to increase with higher blood pressure only in people whose usual systolic blood pressure was 130 mmHg or higher; below this blood pressure threshold, the CVD risk increased with lower blood pressure levels [24]. This relationship has been commonly described as a J-shaped curve, and raises a controversial implication in that, unlike the observations involving incident outcomes, lowering the blood pressure to a level below a certain threshold may prove to be harmful for certain patient groups, such as those who already had CVD previously.

## Pharmacological Interventions to Lower Blood Pressure: Evidence from Tabular Meta-Analysis of Published Results of Randomised Clinical Trials

The contrasting observations between population-based and patient cohorts are difficult to resolve given that observational studies remain prone to residual confounding. In addition, the risk associated with high blood pressure levels seen in observational studies does not necessarily establish raised blood pressure as a modifiable risk factor. Randomised trials investigating the effects of pharmacological agents to lower blood pressure provide evidence for the unconfounded effects of blood pressure–lowering on CVD outcomes and can demonstrate that such treatment can modify the risk associated with elevated blood pressure. In a meta-analysis based on published data from these trials, the effect of lowering systolic blood pressure by 10 mmHg resulted in a proportional reduction in the risk of major cardiovascular events by 20% (95% confidence interval [CI], 17 to 23%) [5•]. There was no significant heterogeneity in the effect according to history of CVD or by baseline systolic blood pressure that ranged from < 130 to ≥ 160 mmHg. Although this and other meta-analyses have established the benefits of blood pressure–lowering, determining the treatment effects among subgroups defined by considering more than one personal characteristic simultaneously is not possible by simply aggregating data from published reports [26, 27]. Thus, to correctly classify people according to their history of CVD as well as by their baseline blood pressure level, individual-level information on these factors is needed.

## The Blood Pressure–Lowering Treatment Trialists’ Collaboration: Recent findings

The Blood Pressure–Lowering Treatment Trialists’ Collaboration (BPLTTC), established in 1995, is a global collaboration of principal investigators and research scientists investigating the effects of blood pressure–lowering drug treatment on CVD and mortality through a series of individual participant-level data (IPD) meta-analyses (www.bplttc.org). The current phase of the BPLTTC has focused on investigating the stratified effects of blood pressure–lowering treatment on major cardiovascular events, other less commonly reported vascular outcomes, and safety outcomes [28]. The collaboration currently includes 52 trials with over 360,000 participants, which forms the largest randomised evidence for blood pressure–lowering treatments to date. In a recent report, the collaboration investigated the benefits of blood pressure–lowering treatment in reducing major cardiovascular outcomes (stroke, myocardial infarction, ischaemic heart disease, or heart failure causing death or requiring hospitalisation) in people with and without prior CVD as well as by baseline blood pressure [29••]. This specific study, based on 48 RCTs with relevant data, included 344,716 participants with a mean age of 65 years.

**Fig. 1:**
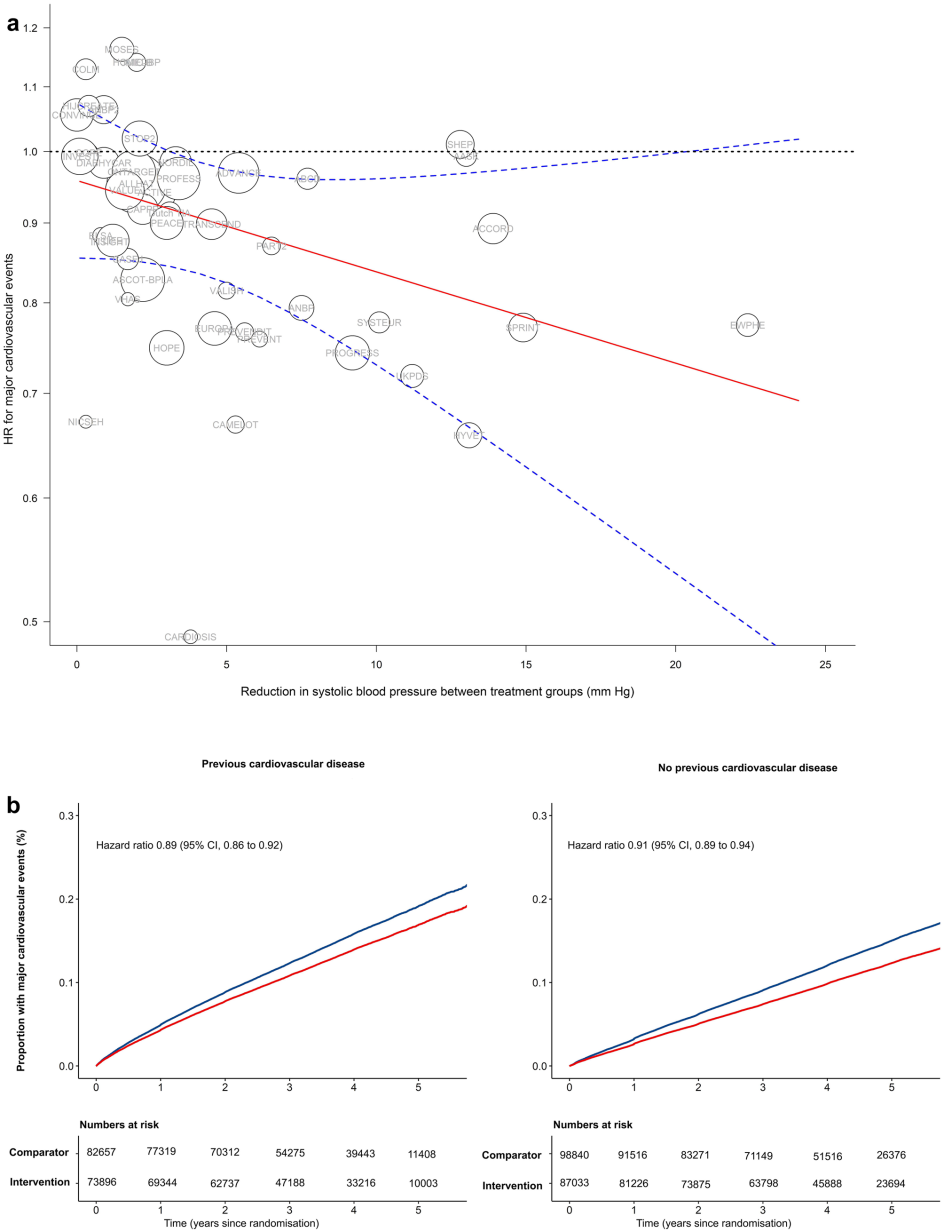
The effect of blood pressure–lowering drug treatment on the risk of major cardiovascular events [29••]. HR, hazard ratio; CI, confidence interval. **A** Intensity of blood pressure reduction in relation to the relative treatment effects on the risk of major cardiovascular events. The centre of the bubbles indicates the HR for each trial, with the size of the bubble inversely proportional to the respective standard error. The solid red line is the fitted regression line; the dashed blue lines indicate 95% confidence interval; and the dashed grey line indicates HR = 1·0. *Excluding the first 12 months after randomisation. **B** Rates of major cardiovascular events per 5 mm Hg reduction in systolic blood pressure, stratified by treatment allocation and cardiovascular disease status at baseline. Major cardiovascular events consisted of fatal or non-fatal stroke, fatal or non-fatal myocardial infarction or ischaemic heart disease, or heart failure causing death or requiring admission to hospital

Nearly a third of the study participants had a known CVD event previously, and there was little difference in the age at baseline between participants with and without a history of CVD. However, baseline systolic/diastolic blood pressure was higher in the latter (157/89 mmHg) than in the former group (146/84 mmHg). Over a median follow-up of 4.2 years, 42,324 participants (12.3%) developed a major cardiovascular event. The achieved blood pressure reduction differed across trials, largely depending on the study design. Since the relative risk reduction was proportional to the magnitude of achieved blood pressure reduction (Fig. 1A), and that treatment effects are known to be largely mediated through blood pressure reduction [25], the relative risk estimates were standardised to a reduction in systolic blood pressure of 5 mmHg.

### Treatment Effects Stratified by History of CVD

Overall, a 5-mmHg reduction in systolic blood pressure lowered the risk of developing the composite outcome of major cardiovascular events by 10% (hazard ratio [HR] (95% CI, 0.90 [0.88 to 0.92]). Similar effects were seen when the components of the composite outcome were examined separately. When the analyses were stratified according to history of CVD, blood pressure–lowering treatment was equally effective in both groups (Fig. 1B). The HR (95% CI) among participants with and without previous CVD was 0.89 (0.86 to 0.92) and 0.91 (0.89 to 0.94), respectively, with no significant heterogeneity in these effects (adjusted *P* for interaction = 1.0). Similar patterns of the results were seen for the effects of treatment in lowering diastolic blood pressure. When expressing treatment effects on absolute disease rates during follow-up, the absolute risk reduction between those with and without previous CVD differed due to a higher risk of future CVD events in those with prior CVD (−0.016 [95% CI −0.019 to −0.012] and −0.022 [95% CI −0.021 to −0.017], respectively; adjusted *P* for interaction = 0.02).

### Effects Stratified by Baseline Blood Pressure in People with and Without a History of CVD

**Fig. 2:**
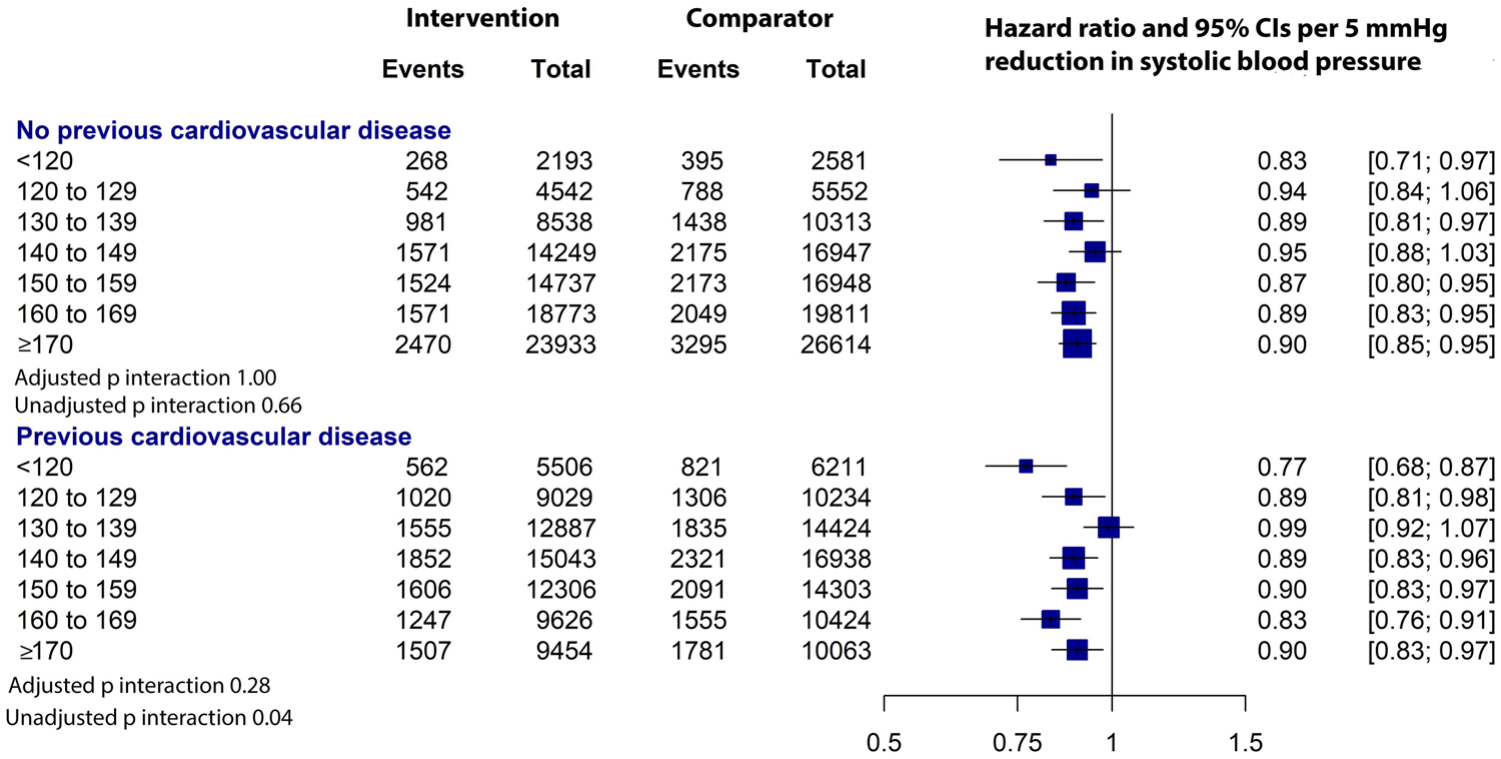
Effects of blood pressure–lowering treatment on major cardiovascular disease events (fatal or non-fatal stroke, fatal or non-fatal myocardial infarction or ischaemic heart disease, or heart failure causing death or requiring admission to hospital), by cardiovascular disease status and systolic blood pressure (mmHg) level at baseline [45••]. The Forest plot shows the hazard ratios (HR) and 95% confidence intervals (CI) per 5 mm Hg systolic blood pressure reduction. Adjusted *p*_interaction_ values were adjusted for multiple testing using Hommel’s method. Unadjusted *p*_interaction_ values were unadjusted for multiple testing

There was a widespread in the baseline blood pressure of trial participants. The systolic blood pressure ranged from < 120 to ≥ 170 mmHg and diastolic blood pressure from < 70 to ≥ 110 mmHg. Among participants without previous CVD (*N* = 186,988), 2.6% had < 120 mmHg systolic and 5.1% with < 70 mmHg diastolic blood pressure; among those with previous CVD (*N* = 157,728), the proportions were 7.5% and 8.8%, respectively. There was no significant heterogeneity in the proportional risk reduction of major cardiovascular events across categories of baseline blood pressure in people with or without prior CVD (Fig. 2), indicating that treatment benefits in people with or without previous CVD were not modified by their baseline blood pressure level. In terms of absolute risk, there was no significant variation in the absolute risk reduction across baseline blood pressure categories in people with or without prior CVD (adjusted *P* for interaction were 0.91 and 1.00, respectively).

### Other Recent Findings: Effects Stratified by Age and by History of Atrial Fibrillation

The BPLTTC has also conducted other IPD meta-analyses, and recently investigated stratified effects of blood pressure–lowering drug treatment by age [30••]. As previously described, epidemiological observations suggest that elevated blood pressure remains a risk factor of CVD in older age ranges [22•]. Even if the relative risks attenuate in older ages, the absolute risk remains high so blood pressure–lowering would still have a beneficial impact in preventing CVD for many in this age group. However, there have been observations suggesting that for those in their ninth decade, ‘low’ or ‘low normal’ blood pressure values have been associated with excess all-cause mortality [31–35]. Hence, there are concerns about interventions that lower blood pressure in this age group particularly if their baseline blood pressure is not high. Evidence for this age group from randomised trials has been limited, and single trials lack power to examine treatment effects in older ages particularly when stratified further by their baseline blood pressure level. In the BPLTTC, lowering of systolic blood pressure by 5 mmHg reduced the risk of major cardiovascular events across ages < 55, 55–64, 65–74, 75–84, and ≥ 85 years (adjusted *P* for interaction = 0.050) [30••]. Although the HR for those aged ≥ 85 years was 0.99 (95% CI 0.87 to 1.12), there was no apparent increase in the risk of all-cause mortality in this age group (HR = 1.00, 95% CI 0.90 to 1.11); further stratification by baseline systolic blood pressure did not indicate any interaction between age and baseline blood pressure (adjusted *P* for interaction = 1.00). While the effect of treatment to reduce CVD was weak in older ages, there was no adverse impact on deaths due to any cause even when their baseline blood pressure was not abnormally elevated.

In a separate study, the BPLTTC also examined any potential heterogeneity in treatment effects according to previous atrial fibrillation [36]. In this study, the beneficial treatment effects were found in people with and without atrial fibrillation at baseline, with HR (95% CI) of 0.91 (0.83 to 1.00) and 0.91 (0.86 to 0.93), respectively (*P* for difference between these groups = 0.9). There was also no impact on all-cause mortality for either subgroups.

## Implications on Determining Treatment Strategies

Many of the prevailing questions surrounding the clinical management of raised blood pressure relate to the blood pressure threshold at which to initiate pharmacotherapy, and, in determining treatment goals, how low should the blood pressure be reduced to.

### To Use or Not to Use Blood Pressure Thresholds for Initiating Treatment?

Pharmacotherapy is generally considered when patients have raised blood pressure, such as in hypertension. Such an approach is typically reflected in clinical guidelines when setting the blood pressure threshold of  ≥ 140/90 mmHg to consider pharmacologic intervention to lower blood pressure. However, evidence from this current study has shown that the beneficial effects of drug treatment are demonstrable even when baseline blood pressure is within the ‘normal’ or ‘high normal’ range. Moreover, the absolute risk reduction did not vary across the blood pressure levels considered in the study, suggesting that baseline level of blood pressure, as a single risk factor, could not discriminate very well the level of CVD risk in the study population. Put differently, there is a substantial proportion of the population whose background risk of CVD is high yet whose blood pressure may not reach the threshold by which pharmacologic treatment could be initiated [20, 37]. Thus, limiting treatment to those with hypertension would mean many of those who are typically ‘normotensive’ but with an elevated background risk of CVD will miss out on the benefits of blood pressure–lowering pharmacotherapy.

On the other hand, considering drug treatment for all has cost implications. Depending on the health care system and service provision, there would be questions around affordability to support such a policy [2]. Moreover, as with taking any medication, there are also potential adverse effects to consider. Hence, it remains important to identify those who are most likely to benefit from blood pressure–lowering drug treatment. Although clinical guidelines largely use a blood pressure–based threshold to inform treatment initiation, others have suggested the use of a CVD risk–based approach, which involves evaluating the background CVD risk using some risk equation calculator, based on several risk factors assessed at baseline and expressed in absolute terms over a given time period. The level of estimated absolute risk will then be used to inform the need for starting pharmacotherapy. This risk-based approach has been reported to be better at identifying those who are most likely to benefit from taking blood pressure–lowering medications than an approach mainly based on a single risk parameter, such as blood pressure [38, 39]. Earlier reports from the BPLTTC have shown that setting thresholds for initiating treatment based on a multivariable risk–based approach would have prevented more events and initiated treatment in fewer people than when using commonly used blood pressure thresholds [40•, 41•]. It has also been suggested that CVD prevention guidelines based on cardiovascular risk strategy will be more cost-effective to implement than when the guidelines are based on blood pressure thresholds [42, 43].

### How Low Should the Blood Pressure be Reduced to?

As mentioned earlier, one of the uncertainties relating to blood pressure–lowering therapy concerns the J-shaped association between blood pressure level and vascular disease risk. If this is true, then there might be a blood pressure threshold associated with the least harm, and reducing blood pressure below this level could potentially cause more harm. However, randomised evidence does not support this notion. As has been described, the benefits of pharmacologic lowering of blood pressure were seen whether or not individuals have had previous CVD and regardless of their baseline blood pressure (and within the blood pressure range commonly observed in the population). Rather than setting a blood pressure target for the treatment to achieve, given that the absolute treatment benefit relates to the magnitude of blood pressure reduction, it may seem more reasonable to ask how much reduction in blood pressure should the treatment strategy aim for.

Physiologically, substantive blood pressure reductions can be achieved in well-controlled studies involving short-term interventions of several weeks or months and relatively compliant participants [44]. However, evidence from long-term effects of commonly trialled drugs and treatment regimens that have informed current practices suggest relatively conservative effects. More intensive blood pressure–lowering interventions have been reported to lower systolic blood pressure by up to 15 mmHg in systolic blood pressure compared to a less intense treatment strategy, giving some guidance on what might be the maximal blood pressure reduction that is feasible to achieve with trialled regimens over several years [45••]. Thus, setting a blood pressure level to target with the pharmacologic treatment strategy might not be feasible for some individuals if they happen to have a very high baseline systolic blood pressure (e.g. 160 mmHg). Yet, for these individuals, the achieved systolic blood pressure reductions of 15 mmHg should still translate into meaningful outcomes even if the achieved level of systolic blood pressure has not fallen below 140 mmHg. Understanding the evidence for the achievable magnitude of blood pressure reduction that can be expected from the trialled drugs and/or regimens should help inform in setting feasible and achievable aims for the clinical management of raised blood pressure.

### Other Considerations for Determining Treatment Strategies

Rather than focusing on modifying the risk factor, the aim of blood pressure–lowering pharmacotherapy should be to improve important health outcomes. By taking this perspective, making treatment decisions primarily based on blood pressure thresholds would be avoided, and individuals whose blood pressure levels may be within the ‘normal’ range, yet whose background risk of CVD is elevated, could still be potentially considered for blood pressure–lowering pharmacotherapy. The wider effects of blood pressure–lowering therapy beyond major vascular outcomes should be considered. Observational studies have reported associations between blood pressure and several vascular conditions which are not frequently included as part of primary outcomes [23•]. There are reports establishing causal associations between elevated blood pressure and increased risk of atrial fibrillation and valvular heart disease [46, 47]. However, evidence from intervention studies remains limited for these other important vascular outcomes. Interestingly, the wider benefits of blood pressure–lowering treatment may extend beyond vascular conditions. In a recent report, the BPLTTC has shown that blood pressure–lowering treatment reduces the risk of diabetes [48••].

Another important consideration in determining treatment strategies is the adverse events linked to the use of blood pressure–lowering medications, particularly when treatment regimens involve intense lowering of blood pressure. These largely non-serious adverse events, such as syncope, hypotension, and falls, were generally time-limited and reversible [49••] and unrelated to baseline blood pressure levels [50]. Previous concerns about the impact of some classes of blood pressure–lowering drugs on cancer risk were also not supported by data from the BPLTTC [51]. These considerations should be part of the discussion between clinicians and their patients in their clinical decision-making.

## Future Directions

Recent studies being led by the BPLTTC have addressed several uncertainties surrounding pharmacotherapy to lower blood pressure, in particular, examining stratified treatment effects according to different patient characteristics. To date, the BPLTTC has reported stratified effects according to a wide range of baseline blood pressure in people with and without previous cardiovascular disease, in middle-aged adults and older individuals, and in those with and without previous atrial fibrillation. Future investigations from the BPLTTC will consider other patient characteristics, such as history of diabetes and renal disease. There is a need for further work to understand treatment effects on other important vascular and non-vascular health outcomes, such as peripheral vascular disease, cognitive function impairment, and bone fractures. Understanding the wide-ranging benefits of blood pressure–lowering treatment may help provide informed discussions on therapeutic options and preferences between patients and clinicians.

Risk-based approaches for determining treatment strategies need further exploration. Some of the multivariable models to estimate an individual’s risk typically use information on personal characteristics, lifestyle, and clinical factors, such as age, sex, smoking status, blood pressure, lipids, and body size, to quantify the probability of developing an event, for example, in the next 10 years. Among the commonly used equations to estimate a patient’s absolute CVD risk at baseline include the Framingham Risk Scores [52], Pooled Cohort Equations [53], SCORE [54], and QRISK [55]. However, data-driven models are increasingly used in health care risk prediction tasks. For example, the use of deep learning algorithms to analyse data extracted from large-scale electronic health records has been reported to have better predictive capacity for vascular health outcomes than various data-driven algorithms [56–58]. Deep learning models may be better at defining at-risk groups which might be relevant in evaluating any heterogeneity of treatment effects that are difficult to identify given conventional analytical methods and when using available trials data. Data-driven approaches may provide novel ways to help identify groups of people with the most and least to gain from blood pressure–lowering treatment for a given age, sex, ethnicity, or health status during a particular period in the person’s life course. However, whether a risk-based approach, based on these novel data-driven models, makes a substantive difference in identifying people who are most likely to benefit from blood pressure–lowering medications compared to commonly used risk assessment tools remains to be seen.

There is also a need to further explore the use of combinations of different drug classes and even new types of drugs to lower blood pressure. A once-daily single pill with low doses of different classes of blood pressure–lowering drugs could be a promising pharmacotherapeutic strategy if it could optimally lower blood pressure as well as cause minimal adverse events [59]. Moreover, it might also be worth exploring novel treatments that could achieve greater blood pressure reduction than can be achieved by current therapies, be these new classes of pharmacologic agents or non-pharmacologic interventions.

## Summary and Conclusions

Pharmacologic lowering of raised blood pressure is effective in the primary and secondary prevention of CVD. These effects are seen across a wide range of baseline blood pressure levels and not limited to those with high values only. However, clinical decision-making to treat raised blood pressure should include several considerations, including the level of blood pressure reduction that is feasible to achieve given the current evidence on the treatment effects of trialled drugs and therapeutic regimens. Conventional approaches that use blood pressure levels to assess the need for pharmacologic interventions to manage raised blood pressure may limit access to such treatment for those whose blood pressure levels do not reach the threshold for ‘abnormally high’ levels yet are at high risk of developing CVD. Alternative strategies to guide treatment decisions could be based on thresholds based on background CVD risk rather than on blood pressure levels. While there are several existing ways to assess background CVD risk, data-driven models, as applied to large-scale health data, may provide novel approaches to estimate the predicted absolute CVD risk at baseline. Future work should further compare and evaluate these risk-modifying approaches to therapy against standard strategies based on blood pressure thresholds in different populations and health care settings. Whichever strategy is used, discussions between patients and clinicians should include treatment effects on other important but less commonly reported vascular and non-vascular disease outcomes, as well as safety outcomes. Finally, future investigations should aim to develop novel pharmacological and non-pharmacological interventions that can achieve greater effects in blood pressure lowering but with minimal, or even less, adverse effects, than can be achieved with existing therapies.
